# Community Knowledge about Water: Who Has Better Knowledge and Is This Associated with Water-Related Behaviors and Support for Water-Related Policies?

**DOI:** 10.1371/journal.pone.0159063

**Published:** 2016-07-18

**Authors:** Angela J. Dean, Kelly S. Fielding, Fiona J. Newton

**Affiliations:** 1 The School of Communication and Arts, The University of Queensland, St Lucia, Queensland, Australia; 2 Cooperative Research Centre for Water Sensitive Cities, Monash University, Clayton, Victoria, Australia; 3 BehaviourWorks, Monash Sustainability Institute, Monash University, Clayton, Victoria, Australia; 4 Faculty of Business and Economics, Monash University, Caulfield, Victoria, Australia; University of Vermont, UNITED STATES

## Abstract

Sustainable approaches to water management require broad community acceptance of changes in policy, practice and technology, which in turn, requires an engaged community. A critical first step in building an engaged community is to identify community knowledge about water management, an issue rarely examined in research. To address this, we surveyed a representative sample of Australian adults (n = 5172). Knowledge was assessed using 15 questions about impact of household activities on waterways, the urban water cycle, and water management. This survey also examined demographics, psychosocial characteristics, exposure to water-related information, and water-related behaviors and policy support. Participants correctly answered a mean of 8.0 questions (Range 0–15). Most respondents knew that household actions can reduce water use and influence waterway health, whereas less than one third correctly identified that domestic wastewater is treated prior to entering waterways, urban stormwater is not treated, and that these are carried via different pipes. Higher water knowledge was associated with older age, higher education and living in non-urban areas. Poorer water knowledge was associated with speaking a language other than English in the home. Garden size, experience of water restrictions, satisfaction, waterway use for swimming, and certain information sources were also associated with knowledge. Greater water knowledge was associated with adoption of water-saving and pollution-reduction behaviors, and support for both alternative water sources and raingardens. These findings confirm the importance of community knowledge, and identify potential subgroups who may require additional targeting to build knowledge and support for water management initiatives.

## Introduction

Ensuring future sustainability of freshwater resources has prompted the need for new paradigms in water management [1, 2]. Traditional water management approaches focused on ensuring adequate water supply, and providing sanitation through wastewater management [3]. However, these traditional approaches have limited sustainability within the context of climate change [4]. Climate change presents a major threat to the world’s freshwater resources, negatively affecting both water availability and water quality [3, 5]. Population growth and urbanisation issues exacerbate these issues [2, 6, 7].

In response to these challenges, new water management paradigms have broadened their consideration of environmental outcomes, focusing on all aspects of the water cycle [3, 4]. These approaches are variously referred to as ‘integrated urban water management’, ‘sustainable urban water management’, ‘water sensitive cities’, or ‘total water cycle management’ [1, 8]. Within these frameworks, water sustainability extends beyond ensuring water supply, to optimising waterway health. This requires management of diverse pollutants that degrade waterways, ensuring they are not introduced into the system at a greater rate than they can be safely absorbed or removed from the system [3]. Pursuing water sustainability within these paradigms requires diverse technological, investment and policy solutions [1–3, 8]. For example, water reuse schemes not only contribute to water availability, but reduce the volume of polluted water entering waterways; water sensitive urban design initiatives and green infrastructure also reduce pollutants entering waterways and generate social benefits [1, 3].

A key element of transitioning to more sustainable water management is building an engaged citizenry [1, 3, 4]. Engagement in water-related issues is multifaceted, incorporating (i) cognitive engagement–knowledge and awareness; (ii) emotional engagement–concern and supportive attitudes; and (iii) behavioral engagement–adoption of civic and household behaviors that promote sustainable water management [9]. Therefore, engaged citizens are those that understand, value, and actively support the necessary changes in technology, investment and policies associated with sustainable water management. Community actions have a significant impact on water demand, water quality, and potentially, the political will of governments to make significant changes to water policy and infrastructure [3, 10]. Community opposition to potable recycled water schemes and the derailing of plans to implement recycled water schemes [11, 12], demonstrate the importance of community support for new water initiatives.

Much research examines determinants and importance of pro-environmental attitudes and behaviors in the community [13–15]. Relatively less research assesses the community knowledge. Therefore, the aim of the current study was therefore to assess Australians’ water-related knowledge, examine social factors associated with this knowledge, and explore the relationship between knowledge and water-related behavior and policy support.

### Importance of knowledge

Knowledge and understanding of water issues in the community is considered a core ingredient of solving water-related problems. Knowledge is central to models of water-related engagement [9], environmental citizenship [16, 17], and environmental literacy [18]. It has been argued that greater knowledge allows community members to contribute to innovation and problem solving [19]. The concept of ‘water literacy’, and other forms of literacy such as health literacy, integrate topic knowledge and the capacity to apply this knowledge to decisions [20, 21]. The literature has not identified specific areas of knowledge considered necessary for adequate water literacy. The emerging emphasis on sustainable water management suggests that key areas of individual-level water knowledge include the urban water cycle and impacts of urbanisation on waterway health via stormwater pollution, in addition to issues related to water demand, supply and treatment [1]. Gauging individual-level knowledge about water is important for a number of reasons. Initiatives that engage with communities are considered more effective when targeted and aligned with the communities’ existing knowledge [22–24]. In addition, identifying strengths and weaknesses in community knowledge about water provides an important foundation for initiatives that aim to increase knowledge.

### Past research on individual-level water knowledge

Despite the importance of assessing knowledge, most existing studies of water-related knowledge are confined to regions of the United States. One of the earliest studies surveyed 1000 residents of California, reporting that most respondents were unaware of water shortages, and had poor understanding of terms describing water sources [25]. More recently, a survey of 17 U.S. states indicated that less than half of the respondents were very familiar with 14 water-related terms (e.g. groundwater) [26]. Another survey, of South Carolina residents, reported that only 28% of respondents could identify the correct definition of a watershed (catchment) [27]. Similarly, a survey of 1000 North Carolina residents demonstrated that only 38% of respondents knew that stormwater flows to the nearest waterway and 30% incorrectly thought that stormwater is treated prior to discharge [28]. Other U.S. studies have examined water-related knowledge at a specific watershed (catchment) level. McDuff and colleagues (2008) surveyed 700 residents and waterway users of the Orange Creek Basin and found that only 36% of respondents had heard of this basin and only 19% could name a major natural feature within the basin, such as a lake or forest [23].

Less research has examined water knowledge in other contexts. One Australian study examined water-related knowledge among 3709 residents of South-East Queensland [29]. It reported mixed results: although 72% of respondents knew that waterways can be damaged by stormwater flows, only 33% could correctly identify that domestic wastewater is treated before entering waterways. Moreover, one in four respondents reported not knowing the specific source of their drinking water [29]. While not a study of water knowledge *per se*, a survey of 1000 Australians found that less than one in five felt that they were very informed about alternative water sources such as recycled water and desalinated water [30].

### Determinants of water-related knowledge

Despite existing surveys reporting examples of poor water-related knowledge, little research has examined determinants of this knowledge. Understanding knowledge and its determinants can be guided by intelligence theories such as Cattel’s Investment Theory. These theories distinguish between two types of intelligence [31, 32]: (i) ‘fluid intelligence’ which incorporates cognitive processing ability and peaks in young adulthood; and (ii)‘crystallized intelligence’ which represents ‘intellect as knowledge’, and incorporates the capacity to retain and apply knowledge. Within this framework, knowledge is not just influenced by educational achievement—and the factors that facilitate educational achievement—but also by diverse life experiences and personal interests that contribute to associative learning [31–33] These pathways align with other research exploring determinants of knowledge. For example, Steel and colleagues [34] examine ‘trans-situational’ and ‘situation-specific’ influences on ocean-related knowledge. Trans-situational factors are those which are important across multiple knowledge domains, such as education and socioeconomic status; situation-specific factors are those which increase topic knowledge, such as personal interest in oceans or visiting the ocean [34, 35].

A range of life experiences—or ‘situation-specific’ factors—could influence water-related knowledge. These include: geographic experience, such as region of residence and experience of drought, or particular rainfall patterns; household context, such as homeownership or the presence of gardens; social experience such as participation in community groups, use of waterways or life satisfaction; and exposure to information. Knowledge about catchments [23] or oceans [34, 36] has been associated with experience of visiting oceans or waterways, reinforcing the potential role of particular experience in developing topic-knowledge. Less research has examined the role of other types of life-experience in contributing to water-related knowledge.

In general, negative life experiences such as emotional stressors or poor life satisfaction have been identified as having the potential to reduce resources available for knowledge acquisition [37]. Elements of social capital, such as participation in community groups may create opportunities for informal learning [38, 39]. There has been limited research, however, examining whether factors such as these influence individual water-related knowledge. Other social factors that may influence knowledge, as suggested by health literacy research, include poorer reading skills, being an immigrant, or speaking a language other than the primary language [40, 41]. In the current research, we aim to examine how these elements of life experience contribute to water-related knowledge.

### Does knowledge influence attitudes and behaviors?

Psychological models of environmental behavior also highlight the importance of knowledge, suggesting that knowledge is a necessary, although not sufficient, ingredient to influence behavior [42]. Research indicates that knowledge can influence diverse pro-environmental behaviors [10, 43, 44]. Importantly, many other factors may also influence pro-environmental behavior, including demographics, social context, psychological factors such as environmental identity and values, and economic factors such as pricing schemes or taxation [10, 14, 38, 45, 46]. Extending beyond individual behaviors, research also suggests a relationship between knowledge and support for policies related to water conservation [47], waterway protection [34, 48, 49], water-sensitive urban design [50], and alternative water sources [51]. This study will extend these findings by examining the relationship between broad-based knowledge about water-related issues and support for water-related behaviors and policies.

### The current study

In summary, there is scant research examining the social determinants of water-related knowledge. The current study addresses this gap by surveying a large, nationally-representative sample of adults residing in Australia, and gauging community knowledge about water-related issues. Australia provides a rich setting to consider community knowledge about water management issues–experience of drought, flooding, and significant population growth necessitate implementation of new water management approaches [52]. Specifically, this study will address the following questions: (i) how does knowledge vary across different water-related issues; (ii) what characteristics are associated with water-related knowledge; and (iii) is water-related knowledge associated with water-related attitudes and behaviors? Study findings will provide actionable insights into the pathways to the acquisition of water-related knowledge and expand our understanding of the relationships between knowledge about water and exposure to sources of water-related information. Taken together, the findings will identify strengths and weaknesses in water-related knowledge, and enable water practitioners to more effectively design and target a range of engagement initiatives, from information campaigns to participatory initiatives.

## Materials and Methods

### Participants and procedure

A total of 5194 adults residing in Australia were recruited by a social research company permission-based, online panel. The sampling frame aimed to ensure a representative sample of the Australian population, based on gender, age, education and state of residence. All eligible panel members were invited to participate via email, and offered the standard compensation (points and entry into a bi-monthly cash prize draw). The 25-minute, online survey was administered during February-March 2014. Ethical clearance was obtained from Monash University and The University of Queensland Ethics Committees prior to study commencement. Participants provided online consent, as approved by the ethics committee.

### Measurement of water-related knowledge

Water-related knowledge was assessed using 15 items about influence of household activities on water quality, catchments and the urban water cycle, and water treatment (Table 1). These items were adapted from a previous study [29], which included items based on what Australian water professionals identified as important for individuals to know about water. Fourteen items were rated on a 5-point Likert scale (1 = ‘*strongly disagree*’ to 5 = ‘*strongly agree*’). A ‘*don’t know*’ option was also included. A Likert-type scale was used in preference to true/false responses, as true/false items can produce high levels of missing data when used to measure objective knowledge of environmental issues. Eight items were framed such that the correct response was ‘agree’/‘strongly agree’; six items were worded such that the correct response was ‘disagree’/ ‘strongly disagree’. Neutral responses (‘don’t know’ or ‘neither disagree or agree’) were coded as incorrect. Finally, one item used a multiple choice response (‘Which of the following options best represents your understanding of what a catchment is?’ Responses options were: (a) The area that retains water like a wetland or a marsh; (b) All of the land area that drains to a specific river or waterway (*correct*); (c) A reservoir that serves as a water source; (d) A small building where water is stored; (e) None of these; (f) Don’t know). A water knowledge index was calculated based on the number of correct responses (Range 0–15).

**Table 1:** Responses to water knowledge statements (population weighted data).

**Knowledge statements**	**% correct (n)**
1. Water conservation actions by householders can significantly reduce the amount of water used in urban areas	73.0% (3789)
2. What individual residents do in their home and garden has consequences for the health of waterways and coastal bays	71.6% (3714)
3. Waterways can be damaged by stormwater flows	67.0% (3478)
4. The fertilizers that individual householders use in their garden can have a negative impact on the health of waterways	66.6% (3456)
5. Planting native plants along a waterway’s bank improves the health of waterways	66.3% (3443)
6. Soil erosion from urban areas does not affect the health of waterways*	59.8% (3105)
7. The pesticides that individual householders use in their garden have no negative impact on the health of waterways*	57.8% (3000)
8. I know where my household drinking water comes from (e.g. dam, groundwater, desalinated water etc.)	53.6% (2779)
9. Waterways can cope easily with large amounts of sediment (i.e. eroded soil suspended in the water)*	52.6% (2728)
10. A catchment is the total land area draining to a specific waterway**	43.9% (2280)
11. The amount of water available for use is finite	40.9% (2123)
12. I know what catchment my household is part of	37.2% (1929)
13. Stormwater from roofs and roads is treated to remove pollutants before entering the waterways*	29.9% (1112)
14. Domestic wastewater and stormwater are carried through the same pipes*	29.2% (1517)
15. Wastewater from domestic bathrooms and laundries receives little or no treatment before entering waterways*	26.0% (1349)

*reverse scored items where the correct response is ‘disagree’ or ‘strongly disagree’.

**Multiple choice question.

### Factors associated with knowledge

#### Demographic and household characteristics

Socio-demographic data collected included: age, sex, highest level of education completed (12 response options, recoded into three yes/no variables: high school only, trade or technical qualification, or university degree), total annual household income bracket before tax (7 response options), and current employment status (9 response options, recoded into four yes/no variables: working, unemployed, retired, studying).

Cultural background was assessed using the following items: Aboriginal or Torres Strait Islander (yes/no); language other than English spoken at home (yes/no); and Ancestry (nine response options each coded yes/no). Household characteristics included: household size (number of people living in household); number of children in household; time resided at current address (years); whether their dwelling was rented or owned, whether the dwelling was an apartment or house; and the size of the garden surrounding their dwelling (no garden, very small <10m^2^, small 11-50m^2^, medium 51-200m^2^, large >200-500m^2^, very large >500m^2^).

#### Geographic characteristics

Geographic location was based on state of residence (each state coded yes/no). The degree of remoteness was classified using the Australian Statistical Geography Standard–Remoteness structure [53]. This classifies postcodes based on distance from major urban centres: major cities, inner regional, outer regional, remote, and very remote. Postcode was also used to calculate regional rainfall statistics, within Australian Bureau of Statistics (ABS) SA4 regions. Rainfall was measured at the weather station closest to the geographic centre of the region (with a bias towards a regions with greater population density). Mean annual rainfall and mean number of days of rain per year were quantified across the 20 year period closest to 2015 [54].

#### Information sources about water

Respondents were asked whether they had seen or heard any information about water from a range of sources in the last six months (yes/no): radio, television, newspapers, online news, water utility newsletter, water utility bill, water utility website, local government newsletter, social media, or no information.

#### Life experience and psychosocial factors

- Experience of water restrictions: two items elicited whether respondents had ever experienced water restrictions (yes/no) and whether they had changed behavior in response to restrictions (yes/no).
- Waterway use: three questions asked how often respondents used their local waterways (defined as creeks, rivers, beaches) to engage in: (i) Regular fishing; (ii) Regular boating (including water-skiing or jet-skiing); and (iii) Regular swimming (including surfing) (5 response options, recoded as yes/no).
- Satisfaction: respondents were asked how satisfied they were with nine life areas (home, employment, finances, safety, community, health, neighborhood, free time, and overall satisfaction). Responses were rated from 0 ‘*Not at all satisfied*’ to 10 ‘*Completely satisfied*’ [55]. The mean of these items created a ‘*Satisfaction’* score (range 0–10, Cronbach’s α = 0.88).
- Community Participation: respondents were asked whether they were a member of, or participated in, any of the following organizations: sporting club, cultural organization, trade union, professional organization, religious organizations, a political party; aid/human rights organization, environmental organization, neighborhood/homeowners association, or other community group [56]. Responses indicating either membership or participation were summed to form a ‘*Participation’* score (range 0–11, Cronbach’s α = 0.79).
- Environmental identity: six items were adapted from past research [13] (e.g. ‘We think of ourselves as an environmentally sustainable household’, ‘There is agreement amongst members of the household that taking action to make the home environmentally sustainable is an important thing to do). Each item utilized a 5-point Likert scale (1 = ‘*strongly disagree*’ to 5 = ‘*strongly agree*’). The mean of these items created a ‘*Household environmental identity’* score (range 1–6, Cronbach’s α = 0.83).

#### Measures of water-related behaviors and policy support

Three variables were created that assessed respondents’ engagement in water-related behaviors:

- Uptake of water saving devices: nine items assessed whether respondents had purchased or installed particular water-saving devices in the home (e.g. water-efficient taps, dual-flush toilets). Response options were: yes, no, or device already in the house. A ‘Water saving device’ percentage score was calculated as the number of ‘yes’ responses (maximum nine), divided by the number of devices *not* already in their home. (Score = #‘yes’ /[9—#‘already in house’], Cronbach’s α = 0.97).
- Use of everyday water-saving strategies: twelve items assessed whether respondents engaged in household water-saving behaviors (e.g. fixing leaks, taking shorter showers). Each item was rated on a 5-point Likert scale (1 = ‘*never’* to 5 = ‘*always*’). The mean of these items was used to create an ‘*Everyday water-saving strategies*’ score (range 1–5, Cronbach’s α = 0.83).
- Pollution-reduction behaviors: seven items assessed whether respondents engaged in pollution reduction behaviors (e.g. preventing animal waste from entering waterways, putting rubbish in the bin). These were rated on a 5-point Likert scale (1 = ‘*never’* to 5 = ‘*always*’). The mean of these items was used to create a ‘*Pollution-reduction behaviors*’ score (range 1–5, Cronbach’s α = 0.69).

Two variables were created that assessed respondents’ support for water-related policies:

- Support for alternative water sources: six items gauged respondents’ support for: use of recycled water use of desalinated water for use of treated stormwater, each for drinking and non-drinking purposes. These items were rated on a 5-point Likert scale (1 = ‘*do not support at all/unwilling*’ through to 5 = ‘*completely supportive/very willing*’). The mean of these items formed a ‘*Support for alternative water sources*’ score (range 1–5, Cronbach’s α = 0.73).
- Support for raingardens: respondents were provided with the statement: “A raingarden is a water-saving garden similar to a regular garden, but designed specifically to capture stormwater from hard surfaces such as driveways, patios and roofs after it rains”. Respondents were asked whether they would be willing to install a raingarden on their property (yes/no), and support a rain garden in their street (yes/no). Positive responses were summed to create a “*Support for Raingardens’* score (range 0–2).

### Statistical analysis

Predictors of water-related knowledge and the relationship between water-related knowledge and water-related attitudes and behaviors were identified using mixed effects linear models. Mixed effects models (also called multilevel models) build on regression techniques by modeling the influence of both fixed and random effects. This allows them to control for the hierarchical nature of the data (i.e. data collected from participants in a particular state may exhibit more relatedness than participants from a different state) [57, 58]. One-way ANOVA indicates significant differences in water-related knowledge across different states (*F* = 10.09, *p*<0.001), reinforcing the importance of controlling for the hierarchical effects of location. Large population datasets typically contain missing data, and include variables that do not occur in balanced proportions. Additional advantages of mixed effects models are that, unlike other regression models, they are unaffected by missing data, and do not require balanced datasets [57, 59]. To address the possibility of under- or over-sampling of certain demographic groups, data were population-weighted based on age and sex, using national data from the Australian Bureau of Statstics [60]

The first stage of analysis examined factors associated with water-related knowledge. As recommended [57], analysis began with a full model, incorporating all demographic, household and geographic characteristics, water information sources, life experience and psychosocial factors (as described in section 2.3) as fixed effects. No interactions were examined. Based on initial model selection using restricted maximum likelihood estimation (REML), a random intercept was included in each model; state was included as random effects to control for the potential clustering effect of location; environmental identity was only included as a random factor to allow generalising of the findings across different levels of household environmental identity [57, 58]. To identify the most optimal fixed structure, each model iteratively removed the least significant factor at each step, using the Akaike Information Criterion (AIC) [61] and maximum likelihood estimation. The final model was refitted using REML [58].

The second stage of analysis examined whether water-related knowledge was associated with the following: (i) Uptake of water-saving devices; (ii) Water-saving behaviors; (iii) Pollution-reduction behaviors; (iv) Support for alternative water sources; (v) Support for raingardens. This analysis focused on whether knowledge was related to behaviors and policy support, and controlled for key factors that may influence environmental these outcomes: demographics (age, sex, education), geographic factors (state of residence, remoteness, rainfall), household factors (homeownership, presence of garden), and life experience/psychosocial factors (household environmental identity, experience of water restrictions, and changing behavior due to water restrictions). Similar to the first stage of analysis, the most optimal fixed structure was identified using the AIC [61] and maximum likelihood estimation. Final models were refitted using REML.

Normality was verified for each model by inspecting histograms of model residuals [58]. Models were also examined to identify potential issues with multicollinearity or heteroscedasticity. All reported models met criteria for normality and did not exhibit problems related to multicollinearity or heteroscedasticity.

## Results

### Sample characteristics

Respondents comprised a representative sample of 5194 Australian adults (mean age 46.9±16.3 years; 50.9% female). The majority of respondents lived in urban centres (77.3%), had qualifications beyond high school (69.1%), and were employed (54.0%) (S1 Table). The most commonly cited sources of water-related information were water utility bills (26.0%) and television (24.4%). More than half the sample (51.3%) reported no exposure to water-related information in the previous six months (S1 Table).

### Water-related knowledge

Based on population weighted data, the mean number of questions correctly answered was 7.76 (SD = 3.99; Range 0–15, 7.76 is equivalent to a score of 52%). Less than one in five respondents scored 80% or above (n = 970), and only 1.4% of respondents (n = 74) answered all items correctly. Almost three quarters of respondents knew that household actions can reduce urban water use and influence the health of waterways, whereas less than one third correctly identified that domestic wastewater is treated prior to entering waterways, urban stormwater is not treated, and that these are carried via different pipes (Table 1).

### Factors associated with water-related knowledge

The mixed model (R^2^ = 0.42) using population weighted data indicates that water-related knowledge was higher in males (*p*<0.001), older respondents (*p*<0.001), those with higher income (*p*<0.001), those currently studying or with greater education (*p*<0.001), and respondents living further away from urban centres (*p*<0.001) (Table 2). A northwest European Ancestry (*p*<0.001) was associated with greater water-related knowledge, whereas having at least one parent born outside Australia (*p*<0.001) and speaking a language other than English at home (*p*<0.01) were associated with lower water-related knowledge. Positive associations were found between garden size (*p*<0.01), experience of water restrictions (*p*<0.001), life satisfaction (*p*<0.001), regular waterway use-swimming (*p*<0.05) and water-related knowledge. Higher levels of water-related knowledge were associated with receiving recent water-related information in utility bills (*p* = 0.05), newsletters from water utilities (*p*<0.05) and local government (*p*<0.01), and social media (*p*<0.05). Respondents reporting no exposure to any water information in the previous six months exhibited poorer water-related knowledge (*p*<0.01) (Table 2). Multilevel models on non-weighted data generated similar findings (S2 Table).

**Table 2:** Final model examining associations with water-related knowledge using multilevel models and population weighted data (AIC original model = 10097.91; AIC final model = 10057.90^a^^,^^b^).

**Fixed factors**	**Descriptives**	**F**	**Standardized coefficient±SE**	**95% CI**
Age	47.0±16.4 (18–85)	180.54***	0.21±0.02	0.18, 0.24
Sex (male)	49.1% (2548)	39.88***	0.15±0.02	0.11, 0.20
Remoteness	See text	15.28***	0.01±0.01	0.03, 0.07
State of residence—Victoria	24.0% (1248)	2.80	-0.08±0.08	-0.03, 0.31
>1 parent born outside Australia	47.7% (2477)	20.16***	-0.12±0.03	-0.17, -0.07
Language other than English at home	18.7% (970)	9.97**	-0.11±0.04	-0.19, -0.04
Ancestry–Northwest Europe	55.5% (2883)	88.75***	0.25±0.3	0.20, 0.30
Ancestry–Sub-Saharan Africa	0.9% (45)	3.69^†^	0.25±0.13	-0.01, 0.50
Income	See S1 Table	5.94***	0.03±0.01	0.01, 0.06
Highest education completed	TAFE 33.9% (1761)	33.18***	0.26±0.03	0.19, 0.32
	Uni 35.1% (1824)		0.10±0.03	0.04, 0.16
Currently studying	5.3% (275)	23.45***	0.26±0.05	0.15, 0.36
Experience of water restrictions	81.7% (4242)	90.16***	0.31±0.03	0.25, 0.38
Waterway use—swimming	16.0% (842)	7.78**	0.09±0.03	0.03, 0.16
Garden size	82.1% (4262) with garden	10.25**	0.04±0.01	0.02, 0.07
Life satisfaction	6.54±1.74 (0–10)	15.07***	0.05±0.01	0.03, 0.08
Participation	1.89±2.43 (0–11)	3.79^†^	0.02±0.01	0.00, 0.05
Water information–utility newsletter	12.7% (658)	5.20*	0.09±0.04	0.01, 0.17
Water information–utility bill	26.0% (1348)	4.10*	0.07±0.04	0.00, 0.14
Water information–local govt. newsletter	9.0% (465)	8.85**	0.13±0.04	0.05, 0.22
Water information–social media	2.7% (138)	4.46*	0.14±0.07	0.01, 0.28
No water information	51.3% (2665)	9.89**	-0.10±0.03	-0.17, -0.04

*p<0.05

**p<0.01

***p<0.001

^†^*p*<0.06.

^a^Variables included in the original model but not retained in the final model as fixed effects: current employment, State of residence (NSW, QLD, SA, WA, TAS), Ancestry (ATSI, Australia-Pacific, SouthEast Europe, SouthEast Asia, Northeast Asia, SouthCentral Asia, Americas, and North Africa-Middle East), Regular waterway use–fishing, Regular waterway use–boating, Number of children, Household size, Duration at current address, Currently renting, Living in apartment, Water information (from newspaper, television, radio, online news, or water website), and rainfall patterns (average rainfall, number of days of rainfall).

^b^Number of cases (observations) included in the final model = 5194.

### Relationships between knowledge and water-related attitudes and behaviors

Five models examined whether water-related knowledge was related to behaviors and policy support using population weighted data. Water-related knowledge was significantly and positively associated with use of everyday water-saving strategies (*p*<0.001), uptake of water-saving devices (*p*<0.001), pollution-reduction behaviors (*p*<0.001), support for alternative water sources (*p*<0.001), and support for raingardens (*p*<0.001) (Table 3). Multilevel models using non-weighted data generated similar findings (S3 Table).

**Table 3:** Final models examining associations between knowledge, and water-related behaviors and policy support, using population weighted data.

	**Use of everyday water-saving strategies**		**Uptake of water-saving devices**		**Pollution-reduction behaviors**		**Support for alternative water sources**		**Support for raingardens**	
**Change in AIC**	4.96		14.21		10.61		13.45		5.16	
**R^2^**	0.36		0.24		0.15		0.19		0.16	
	**F**	**Coefficient±SE**	**F**	**Coefficient±SE**	**F**	**Coefficient±SE**	**F**	**Coefficient±SE**	**F**	**Coefficient±SE**
**Water-related knowledge**	85.21***	**0.13±0.01**	15.93***	**0.06±0.01**	18.08***	**0.07±0.02**	370.67***	**0.30±0.02**	272.38***	**0.26±0.02**
Age	84.94***	0.12±0.01			9.82**	0.05±0.02	9.24**	0.04±0.01	111.85***	-0.16±0.01
Sex (male)	8.57**	-0.07±0.02					23.53***	0.13±0.03		
Education TAFE					5.26**	0.02±0.03			12.13***	0.02±0.03
Uni						-0.08±0.03				0.15±0.03
State—NSW	9.61**	-0.12±0.01	26.94***	-0.22±0.04					3.78	0.10±0.05
State–Victoria			15.55***	-0.24±0.06					9.69**	0.20±0.06
State–Western Australia	9.19**	-0.16±0.05	28.33***	-0.31±0.06			35.96***	0.30±0.05	4.76*	0.15±0.07
State—Tasmania			28.09***	-0.65±0.12						
State—Queensland	5.95*	-0.11±0.04								
State–South Australia					13.13***	0.24±0.07	6.71*	0.14±0.05	8.93**	0.24±0.08
Remoteness	3.56	0.02±0.01			14.42***	0.05±0.01	3.31	-0.02±0.01		
Annual rainfall			8.00**	0.00±0.00	4.09*	0.00±0.00			6.28*	0.00±0.00
Number of days of rain/year			7.76**	0.00±0.00						
Garden	381.13***	0.64±0.03	41.90***	0.24±0.04	66.72***	0.31±0.04			14.87***	0.14±0.04
Renting	217.36***	-0.39±0.03	699.08***	-0.80±0.03	24.13***	-0.16±0.03				
Experience of water restrictions	41.80***	0.09±0.01								
Experience of behavior change during restrictions	38.18***	0.08±0.01	20.19***	0.06±0.01	30.36***	0.08±0.01	17.30***	0.06±0.01	21.61***	0.06±0.01
Environmental identity	187.99***	0.21±0.02	61.61***	0.13±0.01	119.28***	0.20±0.02	83.85***	0.15±0.02	72.17***	0.15±0.02

*p<0.05

**p<0.01

***p<0.001.

## Discussion

To our knowledge, the current study is the first to comprehensively examine factors associated with water-related knowledge among a nationally-representative community sample. Our findings identify strengths and weaknesses in knowledge about water-related issues, and a range of factors associated with this knowledge. These findings provide a basis for information and education initiatives targeting either (i) content areas of poor water-related knowledge, such as wastewater and stormwater treatment (ii) demographic subgroups with poorer levels of water-related knowledge, such as young people and individuals who do not speak English in the home, or (iii) population subgroups who are not accessing suitable sources of water information. The current research also confirmed a relationship between our measure of broad water knowledge and water-related behaviors and policy support.

Similar to other studies [23, 27], the overall level of water-related knowledge was low, with less than one in five respondents correctly answering at least 80% of questions. We observed substantial variation in accurate responses, with many respondents exhibiting high knowledge in some areas, and low knowledge in other areas. Consistent with research indicating that information is more likely to be transmitted and retained if it is relevant [62], our findings reveal higher levels of water-related knowledge about issues directly related to households (such as the impact of household behaviors on water use or waterways), and lower levels of knowledge about issues that households have little control over (such as stormwater or wastewater treatment). Water supply and treatment systems are often ‘invisible’ to households [63] and may be perceived as not relevant [64].

Older respondents and those with greater education exhibited higher water-related knowledge. These findings align with research examining other types of knowledge [23, 34, 65]. Many components of water-related knowledge are likely to be highly correlated with general literacy, such as reading ability [21]; this may, in turn, influence capacity to acquire and retain knowledge in specific areas. Consistent with other research [33, 65], older age was associated with having greater knowledge. Older populations also demonstrate greater engagement in pro-environmental behaviors [66]; data suggests that this is a consequence of greater cumulative exposure to information and experience, which then translates into behavior change [66]. Relevant life experiences could include exposure to drought, maintaining larger gardens, or regular swimming in waterways—all of which were associated with higher knowledge in both of our models. This raises the question of whether educational experiences can provide a ‘short-cut’ to knowledge in younger or less experienced individuals [67].

Certain information sources–newsletters from water utilities or local government, water utility bills, and social media–were associated with greater water-related knowledge. This highlights the potential for information to enhance water-related knowledge, and ultimately, water literacy. The potential for information provided by water utilities to build knowledge may be limited to home owners; those renting or residing in apartments often have water usage covered as part of their rental agreement [68]. Additional channels need to be identified to reach these cohorts of individuals [69]. Different information platforms may vary in their capacity to build knowledge. Our findings indicate that exposure to water-related information via television or radio was not related to greater water-related knowledge. It is possible that active dissemination of information (via newsletters) may be more effective in building knowledge than the use of passive media such as radio or television. A number of studies report that exposure to television was not associated with knowledge [34, 70], even after controlling for cognitive ability [70]. Although sharing information via mass media may represent an appealing [23] and highly-accessed media source, mass media such as television may not provide information of adequate quality, frequency or contextual relevance necessary to build broad-based knowledge. Interestingly, one study reports that compared to commercial television, watching non-commercial (state) television was associated with greater support for recycled water schemes, highlighting the importance of the type and quality of information [71]. It is important to recognise that greater topic knowledge may also increase comprehension and retention of new information [33, 70, 72, 73].

We observed that water-related knowledge was associated with a variety of behaviors and support for policies relevant to sustainable water management. This is consistent with other research linking greater knowledge with adoption of water-related attitudes and behaviors [10, 44, 47, 48], and reinforces the importance of knowledge as a necessary ingredient contributing to policy support or behavior change. Whilst provision of information can increase knowledge and support for policies [74, 75], it is important to recognize that knowledge is not just a product of exposure to information, but is influenced by a range of social factors such as life experience and personal relevance. Individuals with poor topic knowledge may also exhibit characteristics such as poor information-processing skills or low personal interest in the topic which reduces the likelihood of information detection or retention. As such, engagement initiatives that provide information only—without addressing the broader social context or actively targeting disengaged subgroups—may not generate meaningful changes in behaviors or policy support [9, 76].

Knowledge may influence household or civic behaviors via many pathways–not only by raising awareness or concern. For example, an individual with poor water-related knowledge may: (i) avoid seeking advice about water due to shame or poor issue awareness; (ii) have difficulty processing information, which may limit engagement with water organisations; or (iii) avoid informal conversations about water, limiting informal information sharing or activating social norms about water use [77]. Knowledge about how to act (procedural knowledge) or the effectiveness of actions (effectiveness knowledge) may have a stronger influence on environmental behavior than general awareness (declarative knowledge) [42, 78]. Similarly, experiential and active learning of skills may generate greater change in behavior than passive acquisition of knowledge [78]. The concept of water literacy–with its focus on processing information, acquiring knowledge and applying knowledge to decisions–allows us to recognize the importance of different types of knowledge and the importance of life experience in acquiring and retaining knowledge [40, 42]. The current study aims to build our understanding of the concept of water literacy.

These findings have a number of practical implications for water practitioners and information providers. It is important to recognize that knowledge is not binary, but varies in depth and breadth across issues–individuals may be well informed on some water issues, but poorly informed on others. When planning engagement or education initiatives, it is important not to assume pre-existing knowledge, and to make information relevant for the target group. Poor understanding of words like ‘catchment’ are a reminder to minimize use of jargon and technical terms when engaging with communities. Identifying factors associated with poorer water-related knowledge may facilitate better targeting of certain community sub-groups for information or engagement-focused campaigns. It remains unclear whether solely focusing on knowledge improvements would translate into increased uptake of behaviors in these groups. Although knowledge and literacy can be cultivated [21], certain target groups may require more intensive interventions to create meaningful engagement.

### Limitations

This study has a number of limitations. Our assessment of water-related knowledge focused on a select set of issues. Although the items we utilized do not represent the definitive content of water-related knowledge, these items were identified as important by members of the water industry. Our use of Likert-scales to rate knowledge, selected to prevent participants ‘feeling examined’, may have over-estimated knowledge, due to response bias, where certain individuals are more inclined to indicate agreement. Measurement techniques such as multiple-choice questions would minimise this issue. Open-ended questions, while avoiding some of the problems with Likert scales, appear to generate inadequate data quality to assess knowledge of complex concepts [79]. Little research has examined the strengths and weaknesses of different approaches to assessing knowledge, especially using online surveys. To build our understanding of water literacy, future research should explore optimal methods for assessing both knowledge about water, and how individuals apply this knowledge to decision making. While our analysis considered an important issue that influences effectiveness of community engagement—knowledge and individual characteristics—it is important to recognize that organizational factors such as trust in institutions can influence effectiveness of engagement [80]. Finally, our research was cross-sectional in nature, making it difficult to fully address causality of these relationships. Future research should evaluate the effectiveness of knowledge-building interventions on policy support and broader engagement.

## Conclusions

This is the first nationally-representative study to examine water-related knowledge and its determinants. Study findings highlight a range of content areas where individuals have poor knowledge, especially regarding management of stormwater and wastewater. Identifying demographic and psychosocial characteristics associated with poor water-related knowledge, provide a foundation for further initiatives or campaigns aiming to build knowledge. Importantly, water-related knowledge was associated with a series of behaviors and support for policies. Our findings demonstrate the value of considering knowledge when building support for sustainable water management initiatives.

## Supporting Information

S1 Table Respondent characteristics for all factors included in original model examining factors associated with knowledge. (DOCX)

S2 Table Final model examining associations with water-related knowledge using multilevel models, without population weights (AIC original model = 9858.83; AIC final model = 9817.35^a,b^). (DOCX)

S3 Table Final models examining associations between knowledge, and water-related behaviors and policy support, without population weights. (DOCX)
