# Supplementary material for: Community Knowledge about Water: Who Has Better Knowledge and Is This Associated with Water-Related Behaviors and Support for Water-Related Policies?
Source: PLoS One. 2016 Jul 18;11(7):e0159063. doi: 10.1371/journal.pone.0159063 (PMC4948862; doi:10.1371/journal.pone.0159063)
Supplement: S1 Table — (DOCX) [file pone.0159063.s001.docx]

**S1 Table:** Respondent characteristics for all factors included in original model examining factors associated with knowledge

| **FIXED FACTOR** | **% (n) or**  **Mean±SD (Range)** |
| --- | --- |
| **Age (years)** | 47.0±16.4 (18-85) |
| **Gender (% female)** | 50.9% (2645) |
| **Household gross annual income** Less than $20,000 | 7.5% (389) |
| $20,000 - $40,000 | 18.1% (940) |
| $40,000 - $60,000 | 15.5% (803) |
| $60,000 - $80,000 | 12.8% (667) |
| $80,000 - $100,000 | 11.8% (612) |
| $100, 000 - $150,000 | 14.2% (739) |
| $150,000 or more | 7.3% (381) |
| **Highest level of education completed** High school only | 30.9% (1607) |
| Trade qualification / Diploma | 33.9% (1761) |
| University | 35.1% (1824) |
| **Employment status** Employed | 54.0% (2804) |
| Unemployed | 5.7% (297) |
| Studying | 5.3% (275) |
| **Remoteness** Major City | 77.3% (4016) |
| Inner Regional | 15.7% (816) |
| Outer Regional | 6.4% (332) |
| Remote | 0.5% (24) |
| Very Remote | 0.1% (6) |
| **State** New South Wales/Australia Capital Territory | 36.3% (1883) |
| Queensland | 19.9% (1035) |
| South Australia | 7.6% (395) |
| Tasmania | 2.3% (118) |
| Victoria | 24.0% (1248 |
| Western Australia | 9.5% (492) |
| **Rainfall** Average annual rainfall (mm) | 842.0±294.1 (257.3-1982.3) |
| Number of days per year | 87.1±15.0 (29.4-167.7) |
| **Ancestry (> one response permitted)** ATSI | 1.6% (82) |
| Australia-Pacific | 33.3% (1730) |
| Northwest Europe | 55.5% (2883) |
| South & East Europe | 11.1% (578) |
| North Africa/Middle East | 1.5% (78) |
| SouthEast Asia | 6.5% (340) |
| NorthEast Asia | 4.9% (255) |
| Southern & Central Asia | 3.2% (166) |
| The Americas | 1.6% (81) |
| Sub-Saharan Africa | 0.9% (45) |
| **≥1 parent born overseas** | 47.7% (2477) |
| **Speaking language other than English at home** | 18.7% (970) |
| **Household size** | 2.76±1.36 (range 1-9) |
| **Currently renting home** | 30.3% (1576) |
| **Has garden** | 82.1% (4262) |
| **Garden size** No garden | 17.9% (931) |
| Very small <10m^2^ | 8.8% (456) |
| Small 11-50m^2^ | 14.8% (771) |
| Medium 21-200m^2^ | 28.8% (1494) |
| Large >200-500m^2^ | 16.7% (866) |
| Very large >500m^2^ | 13.0% (675) |
| **Time lived at current address (years)** | 10.6±11.1 (1-72) |
| **Experience of water restrictions** | 81.7% (4242) |
| **Experience of changing behavior during restrictions** | 53.6% (2784) |
| **Waterway user – fishing** | 7.9% (410) |
| **Waterway user – boating** | 5.4% (279) |
| **Waterway user - swimming** | 16.0% (842) |
| **Water information** Water utility bill | 26.0% (1348) |
| Television | 24.4% (1266) |
| Newspapers | 18.3% (950) |
| Water utility newsletter | 12.7% (658) |
| Radio | 10.0% (521) |
| Local government newsletter | 9.0% (465) |
| Online news | 7.9% (410) |
| Water utility website | 6.6% (341) |
| Social media | 2.7% (138) |
| No information about water | 51.3% (2665) |
| **Life satisfaction** | 6.54±1.74 (0-10) |
| **Community participation** | 1.89±2.43 (0-11) |
| **Household environmental identity** | 3.61±0.70 (1-5) |
| **Experience of water restrictions** | 81.7% (4242) |
| **Experience of behaviour change during restrictions** | 53.6% (2784) |
