# Supplementary material for: Community Knowledge about Water: Who Has Better Knowledge and Is This Associated with Water-Related Behaviors and Support for Water-Related Policies?
Source: PLoS One. 2016 Jul 18;11(7):e0159063. doi: 10.1371/journal.pone.0159063 (PMC4948862; doi:10.1371/journal.pone.0159063)
Supplement: S2 Table — (DOCX) [file pone.0159063.s002.docx]

**S2 Table: Final model examining associations with water-related knowledge using multilevel models, without population weights (AIC original model=9858.83; AIC final model=9817.35^a,b^)**

| **Fixed factors** | **Descriptives** | **F** | **Standardized coefficient ±SE** | **95% CI** |
| --- | --- | --- | --- | --- |
| Age | 47.0±16.4 (18-85) | 197.31^***^ | 0.22±0.02 | 0.19, 0.25 |
| Sex (male) | 49.1% (2548) | 40.04^***^ | 0.15±0.02 | 0.11, 0.20 |
| Remoteness | See text | 15.70^***^ | 0.05±0.01 | 0.03, 0.08 |
| >1 parent born outside Australia | 47.7% (2477) | 26.88^***^ | -0.13±0.03 | -0.18, -0.08 |
| Language other than English at home | 18.7% (970) | 8.84^**^ | -0.11±0.04 | -0.18, -0.04 |
| Ancestry – Northwest Europe | 55.5% (2883) | 89.53^***^ | 0.25±0.03 | 0.20, 0.31 |
| Ancestry – Sub-Saharan Africa | 0.9% (45) | 2.78 | 0.25±0.15 | -0.04, 0.55 |
| Income | See S1 Table | 5.91^*^ | 0.03±0.01 | 0.01, 0.06 |
| Highest education completed | TAFE 33.9% (1761) | 38.81^***^ | 0.15±0.03 | 0.09, 0.20 |
|  | Uni 35.1% (1824) |  | 0.28±0.03 | 0.22, 0.34 |
| Currently studying | 5.3% (275) | 26.06^***^ | 0.31±0.06 | 0.19, 0.43 |
| Experience of water restrictions | 81.7% (4242) | 79.94^***^ | 0.30±0.03 | 0.23, 0.37 |
| Waterway use - swimming | 16.0% (842) | 5.60^*^ | 0.08±0.03 | 0.01, 0.14 |
| Garden size | 82.1% (4262) with garden | 9.63^**^ | 0.04±0.01 | 0.01, 0.06 |
| Life satisfaction | 6.54±1.74 (0-10) | 14.71^***^ | 0.05±0.01 | 0.02, 0.08 |
| Water information –utility newsletter | 12.7% (658) | 7.60^**^ | 0.11±0.04 | 0.03, 0.19 |
| Water information –utility bill | 26.0% (1348) | 3.85^†^ | 0.07±0.04 | 0.00, 0.14 |
| Water information – local govt. newsletter | 9.0% (465) | 9.80^**^ | 0.14±0.04 | 0.05, 0.22 |
| No water information | 51.3% (2665) | 16.98^***^ | -0.13±0.03 | -0.19, -0.07 |

^*^*p*<0.05; ^**^*p*<0.01; ^***^*p*<0.001, ^†^*p*=0.050

^a^Variables included in the original model but not retained in the final model as fixed effects: current employment, State of residence (NSW, QLD, SA, WA, VIC, TAS), Ancestry (ATSI, Australia-Pacific, SouthEast Europe, SouthEast Asia, Northeast Asia, SouthCentral Asia, Americas, and North Africa-Middle East), Regular waterway use–fishing, Regular waterway use–boating, Number of children, Household size, Duration at current address, Currently renting, Living in apartment, Participation, Water information (from newspaper, television, radio, online news, water website, or social media), and rainfall patterns (average rainfall, number of days of rainfall).

^b^Number of cases (observations) included in the final model = 5194
