# Supplementary material for: Community Knowledge about Water: Who Has Better Knowledge and Is This Associated with Water-Related Behaviors and Support for Water-Related Policies?
Source: PLoS One. 2016 Jul 18;11(7):e0159063. doi: 10.1371/journal.pone.0159063 (PMC4948862; doi:10.1371/journal.pone.0159063)
Supplement: S3 Table — (DOCX) [file pone.0159063.s003.docx]

**S3 Table: Final models examining associations between knowledge, and water-related behaviors and policy support, without population weights**

|  | **Use of everyday water-saving strategies** | | **Uptake of water-saving devices** | | **Pollution-reduction behaviors** | | **Support for alternative water sources** | | **Support for raingardens** | |
| --- | --- | --- | --- | --- | --- | --- | --- | --- | --- | --- |
| **AIC Δ** | 3.36 | | 10.55 | | 7.91 | | 8.98 | | 8.17 | |
| **R^2^** | 0.36 | | 0.22 | | 0.14 | | 0.19 | | 0.15 | |
|  | **F** | **Coefficient±SE** | **F** | **Coefficient±SE** | **F** | **Coefficient±SE** | **F** | **Coefficient±SE** | **F** | **Coefficient±SE** |
| **Water-related knowledge** | **102.61^***^** | **0.14±0.01** | **17.98^***^** | **0.06±0.01** | **16.82^***^** | **0.07±0.02** | **312.90^***^** | **0.29±0.02** | **256.76^***^** | **0.26±0.02** |
| Age | 61.22^***^ | 0.10±0.01 |  |  | 5.55^*^ | 0.04±0.02 | 8.04^**^ | 0.04±0.01 | 131.57^***^ | -0.17±0.01 |
| Sex (male) | 11.03^**^ | 0.08±0.02 |  |  |  |  | 25.91^***^ | 0.14±0.03 |  |  |
| Education TAFE |  |  |  |  | 6.62^**^ | 0.03±0.03 | 4.17^*^ | 0.04±0.03 | 9.85^***^ | 0.04±0.03 |
| Uni |  |  |  |  |  | -0.09±0.03 |  | 0.10±0.03 |  | 0.14±0.03 |
| State - NSW | 16.99^***^ | -0.14±0.03 | 34.09^***^ | -0.22±0.04 | 6.62^*^ | -0.14±0.06 | 8.62^**^ | -0.10±0.03 |  |  |
| State – Victoria |  |  | 16.39^***^ | -0.23±0.06 | 13.77^***^ | -0.22±0.06 | 5.31^*^ | -0.08±0.04 |  |  |
| State – Western Australia | 9.20^**^ |  | 25.50^***^ | -0.28±0.05 | 6.10^*^ | -0.17±0.07 | 30.51^***^ | 0.28±0.05 |  |  |
| State - Tasmania |  |  | 26.20^***^ | -0.59±0.12 |  |  |  |  |  |  |
| State - Queensland | 8.75^**^ | -0.11±0.04 |  |  | 8.26^**^ | -0.17±0.06 |  |  |  |  |
| State – South Australia |  |  |  |  |  |  |  |  |  |  |
| Remoteness | 5.48^*^ | 0.03±0.01 |  |  | 8.84^**^ | 0.04±0.01 |  |  |  |  |
| Annual rainfall |  |  | 6.00^*^ | -0.06±0.03 |  |  |  |  |  |  |
| Number of days of rain/year |  |  | 4.02^*^ | 0.05±0.02 |  |  |  |  |  |  |
| Garden | 391.29^***^ | -0.65±0.03 | 37.20^***^ | 0.23±0.04 | 65.64^***^ | 0.31±0.04 |  |  | 10.40^**^ | 0.12±0.04 |
| Renting | 235.06^***^ | -0.42±0.03 | 645.92^***^ | -0.77±0.03 | 26.18^***^ | -0.016±0.03 |  |  |  |  |
| Experience of water restrictions | 29.13^***^ | 0.08±0.01 |  |  |  |  | 4.53^*^ | 0.04±0.02 | 5.79^*^ | 0.04±0.02 |
| Experience of behavior change during restrictions | 40.58^***^ | 0.08±0.01 | 18.27^***^ | 0.06±0.01 | 32.95^***^ | 0.08±0.01 | 18.63^**^ | 0.06±0.01 | 23.79^***^ | 0.07±0.01 |
| Environmental identity | 198.46^***^ | 0.20±0.01 | 67.14^***^ | 0.13±0.02 | 142.76^***^ | 0.20±0.02 | 59.73^***^ | 0.14±0.02 | 84.98^***^ | 0.15±0.02 |

^*^*p*<0.05; ^**^*p*<0.01; ^***^*p*<0.001
